# Holmium-166 radioembolisation dosimetry in HCC

**DOI:** 10.1007/s00259-024-06940-2

**Published:** 2024-10-29

**Authors:** Margot T. M. Reinders, Arthur J. A. T. Braat, Karel J. van Erpecum, Joep de Bruijne, Rutger C. G. Bruijnen, Dave Sprengers, Rob de Man, Erik Vegt, Jan N. M. IJzermans, Sjoerd G. Elias, Marnix G. E. H. Lam, Maarten L. J. Smits

**Affiliations:** 1 Department of Radiology & Nuclear Medicine, Utrecht University - University Medical Centre Utrecht, P.O. Box 85500 100, 3500 GA Utrecht, the Netherlands; 2 Department of Gastroenterology & Hepatology, Utrecht University - University Medical Centre Utrecht, P.O. Box 85500 100, 3500 GA Utrecht, the Netherlands; 3 Department of Gastroenterology & Hepatology, Erasmus MC-University Medical Centre Rotterdam, P.O. Box 2040, 3000 CA Rotterdam, the Netherlands; 4 Department of Radiology & Nuclear Medicine, Erasmus MC-University Medical Centre Rotterdam, P.O. Box 2040, 3000 CA Rotterdam, the Netherlands; 5 Department of Surgery, Erasmus MC-University Medical Centre Rotterdam, P.O. Box 2040, 3000 CA Rotterdam, the Netherlands; 6 Julius Centre for Health Sciences and Primary Care, Utrecht University - University Medical Centre Utrecht, P.O. Box 85500 100, 3500 GA Utrecht, the Netherlands

**Keywords:** Holmium, Radioembolisation, Hepatocellular carcinoma, Dosimetry, Personalised treatment planning, Biodistribution

## Abstract

**Purpose:**

To evaluate dosimetry, dose–response and dose-toxicity relationships for holmium-166 (^166^Ho) radioembolisation in patients with hepatocellular carcinoma (HCC).

**Methods:**

Thirty-one patients with hepatocellular carcinoma were included in the HEPAR Primary study (NCT03379844, registered on December 20th, 2017) and underwent ^166^Ho-microspheres radioembolisation. Linear mixed models assessed the association between tumour absorbed doses and response based on mRECIST both on tumour and patient level. Preliminary tumour absorbed dose thresholds were estimated based on predictive value. Linear regression models assessed the association between non-tumour absorbed dose and Common Terminology Criteria for Adverse Events version 4.03.

**Results:**

Median tumour absorbed dose (tumour level) was 95.5 Gy (range 44—332 Gy). Median non-tumour absorbed dose based on whole liver volume was 19 Gy (range 3 – 48 Gy) and based on target liver volume was 30 Gy (range 13 – 54 Gy). There was a significant association between non-tumour absorbed dose and toxicity. Tumours with partial response/complete response (PR/CR, responders) received a 41% higher absorbed dose than tumours with progressive disease/stable disease (PD/SD, non-responders) (95%CI: 2%-93%, *p* = 0.04). A predictive value of 90% for tumour response was observed at a tumour absorbed dose threshold of 155 Gy, 100% predictive value was achieved at 184.5 Gy.

**Conclusion:**

This study confirms a positive relationship between tumour absorbed dose and response and between non-tumour absorbed dose and toxicity. Dose thresholds found in this study can serve as a basis for personalized dosimetry in HCC patients treated with ^166^Ho-microspheres.

**Supplementary Information:**

The online version contains supplementary material available at 10.1007/s00259-024-06940-2.

## Introduction

Radioembolisation is based on the intra-arterial injection of radioactive microspheres that lodge in the vessel(s) supplying the tumour(s) and irradiate the tumour from within. A scout dose of holmium-166 (^166^Ho)-microspheres (QuiremScout™, Terumo) can be used for treatment simulation instead of the commonly used technetium-99m (^99m^Tc)-macroaggregated albumin particles (^99m^Tc-MAA) [1]. After full assessment of the distribution of the scout dose, a therapeutic dose of ^166^Ho-microspheres can be administered (QuiremSpheres™, Terumo).

Up to now, patients treated with ^166^Ho-microspheres radioembolisation received a standard amount of activity corresponding to an average perfused liver absorbed dose of 60 Gy (i.e. single compartment dosimetry) [2]. Currently, there is a paradigm shift from such a ‘one-size-fits-all’ approach towards individualized dosimetry, using the distinction between tumour and non-tumour absorbed doses. The population of patients with hepatocellular carcinoma (HCC) especially calls for such an approach, as most patients suffer from underlying liver disease [3]. For individualised treatment planning, a reliable distribution predictor is needed as well as thresholds for tumour absorbed dose (i.e. the minimal absorbed dose that will lead to a high chance of tumour response) and non-tumour dose (i.e. the maximum safe absorbed dose to the healthy liver) [4, 5].

Dose thresholds are influenced by many factors, among which type of microsphere, type of tumour, clinical scenario and underlying liver disease. Tumour and non-tumour absorbed dose thresholds were previously studied for ^166^Ho-radioembolisation in patients with colorectal cancer [4, 5]. The current work is the first to assess dose thresholds for HCC patients treated with ^166^Ho-microspheres radioembolisation.

In this study, the association between tumour absorbed dose and outcome in terms of tumour and patient level response and survival were evaluated and the association between non-tumour absorbed dose and toxicity were assessed. Optimal tumour and non-tumour tissue absorbed dose thresholds for patients with HCC treated with ^166^Ho-microspheres radioembolisation were investigated as a preliminary basis for individualised treatment planning.

## Methods

### Study design

The HEPAR Primary study was a multicentre, non-randomized, interventional, open label, phase II study and included 31 patients with HCC who were treated with ^166^Ho-microspheres radioembolisation [6]. The primary endpoint was rate of unacceptable toxicity defined as grade 3 hyperbilirubinemia in combination with low albumin and/or ascites in the absence of disease progression (CTCAE v.4.03) or treatment-related serious adverse events (SAEs). Secondary endpoints included overall toxicity, response, survival, change in alpha-fetoprotein and quality of life. Patients were included based on the following main inclusion criteria: ≥ 18 years of age, life expectancy of at least six months, diagnosis of HCC according to criteria of the American Association for the Study of Liver Disease (AASLD) with measurable lesion(s) based on modified response evaluation criteria in solid tumours criteria (mRECIST) [7], liver-dominant disease (maximum five lung nodules all ≤ 1.0 cm, and mesenteric or portal lymph nodes all ≤ 2.0 cm), no curative treatment options, Child–Pugh (CP) ≤ B7, and a good performance status (Eastern Cooperative Oncology Group, ECOG 0–1) [8]. Patients with previous or current treatment with radioembolisation or portal vein thrombosis (PVT) of the main branch were excluded [9]. Lack of activity in the thrombus was an exclusion criterion. For dosimetry purposes the tumour thrombus was included in the tumour volume. If scout dose imaging revealed a projected lung shunt > 30 Gy or uncorrectable extrahepatic deposition of scout dose activity, patients could not further continue the study. The study was approved by the Medical Ethics Committee of the University Medical Centre Utrecht and the institutional radiation protection committee and was performed in accordance with Good Clinical Practice and the declaration of Helsinki. All patients provided written informed consent prior to participation. Safety analyses were presented to a Data Safety Monitoring Board, consisting of one biostatistician, one oncologist, one nuclear medicine physician and one interventional radiologist. The study was registered with ClinicalTrials.gov, NCT03379844.

### Patient assessment

Laboratory and clinical examinations were performed during all follow-up visits (three and six weeks, three and six months after treatment). Per protocol, both laboratory and clinical toxicities were evaluated based on Common Terminology Criteria for Adverse Events (CTCAE) version 4.03. New and highest grade of toxicity during follow-up was used. Pre-existing toxicities were not taken into account unless they deteriorated after treatment. Unacceptable toxicity was defined in the protocol as hyperbilirubinemia grade 3 or higher in combination with low albumin and ascites in the absence of disease progression. Toxicity was also scored using the relative changes in bilirubin, albumin and International Normalised Ratio (INR) and the changes in model for end-stage liver disease (MELD), Child–Pugh (CP) and ALBI scores three months after treatment compared to baseline. Liver toxicity was defined as grade 3–5 in bilirubin and/or albumin levels and/or presence of ascites according to CTCAE.

During follow-up, MRI was acquired three and six months after treatment to assess tumour response. Tumour response (i.e. target lesion response and overall response) was scored by two experienced and independent radiologists, using mRECIST, dividing response into four categories: progressive disease (PD), stable disease (SD), partial response (PR) or complete response (CR) [7]. In case of discordance between the radiologists, a third radiologist decided on the response category.

### Treatment planning

Treatment planning was performed aiming for an average absorbed dose of 60 Gy in the target volume, according to the MIRD method as described in the instructions for use for ^166^Ho-microspheres [10]. The target volume consisted of two or more segments, one lobe or the whole liver. The dose was calculated using the following formula: Injected activity (MBq) = target volume weight (in g) × 3.78 (MBq/g). Prescribed activity was not corrected for lung shunt fraction.

### Image acquisition

Baseline imaging included a contrast enhanced CT-scan (Philips iCT) and an MRI-scan (Philips Ingenia 1.5T, release 5.3) to assess vasculature and tumour involvement. CT liver protocol included imaging after iodine contrast injection (flow speed 5 mL/s) in arterial, porto-venous and late phase. MRI protocol consisted of T1 in expiration, T2 free breathing, multi-echo fast field echo (mFFE) and dynamic series with contrast (0.1 mL Gadobutrol/kg; flow speed of 1 mL/s). After receiving a scout dose of ^166^Ho-microspheres scintigraphy was performed. First a static planar image was acquired for 5 min. (matrix 256 × 256 with medium-energy low-penetration (MELP) collimator) to calculate lung shunt fraction. Then quantitative SPECT was acquired (Siemens, Symbia T16 slices, 60 frames; 15 s/frame; matrix 128 × 128; MELP collimator; energy windows 81 and 140 keV (15%), 118 and 170 keV (12%)) followed by a low-dose CT (5 mm slices; 110 kV).

Within 24 h after treatment a quantitative MRI-scan was acquired (same T1 and T2 scans with and without breath hold, but no dynamic series). Due to detector dead time caused by the abundance of γ-photons, 3–5 days after treatment a post-therapeutic SPECT-CT was acquired, using the same protocol as after the scout dose.

### Image analysis

Imaging data for both SPECT-CT and MRI was analysed using QSuite version 2.0 provided by Quirem Medical B.V. First, volumes of interest (VOI) were delineated on baseline MRI for the whole liver, target liver, tumour(s) and healthy liver (tumours subtracted from the whole liver volume). It was assumed that no interval progression took place between pre-treatment imaging and treatment. Quantitative SPECT scans were reconstructed with Utrecht Monte Carlo Simulation [11], matrix size 128 × 128x81, resolution 4.80 × 4.80x4.80 mm. SPECT scans were rigidly co-registered with low dose CTs (matrix 512 × 512x78 and resolution 1.27 × 1.27x5 mm) that were acquired at the same time. SPECT counts in the delineated volume of treated liver (calibration VOIs) were used for local dose deposition calculation method (using the assumption that all dose is deposited within the voxel of origin) based on administered activity per patient (MBq). All registrations were visually checked and manually adjusted if needed. Tumours smaller than 5 mL were excluded from dose measurements. Finally, dose evaluation was based on dose reconstructions and delineations, resulting in average absorbed dose per VOI (Gy). Furthermore, a mean tumour absorbed dose per patient was calculated using a weighted average based on volume and number of lesions. The non-tumour absorbed dose was separately calculated for the target volume and the whole liver, based on the activity in the target volume minus the activity in the tumours and based on the activity in the whole liver volume minus the activity in the tumours, respectively.

### Statistical analysis

Descriptive statistics were used to provide an overview of patient and treatment data. The association between non-tumour tissue absorbed dose (independent continuous variable) and percent change in laboratory parameters (dependent continuous variable) was assessed using ordinary linear regression models. Furthermore, the association was investigated between non-tumour tissue absorbed dose (independent continuous variable) and CTCAE laboratory toxicity (dependent categorical variable). As AEs grade 3 and higher were considered of clinical significance, both laboratory and clinical adverse events were dichotomized in grade 0–2 vs. 3–5. On these dichotomized variables logistic regression analyses were performed. Because patients with progressive disease may have impaired liver function regardless of radioembolisation treatment, toxicity analyses were adjusted for patient response (PD/SD versus PR/CR). They were also adjusted for presence of liver cirrhosis at baseline, as the hepatic cells of cirrhotic patients may have an altered reaction to radiation compared to patients without cirrhosis. Logistic regression analysis of the dichotomized adverse events (grade 0–2 vs. grade 3–5) for both laboratory as well as clinical events was represented by the odds ratio (Supplemental Table S1).

A linear mixed effect model was used to investigate the relationship between tumour absorbed dose (dependent continuous variable, log-transformed to conform model assumptions) and best response during follow up based on mRECIST (independent categorical variable), considering clustering of multiple tumours within patients using a random intercept. As the unit of observation was tumour and not patient, mixed models were used to allow for clustering within patients. Models were compared using Akaike’s Information Criterion and were adjusted for non-tumour dose and presence of extrahepatic disease at baseline. Patients that received a higher non-tumour dose may have had more damage to hepatic cells resulting in liver dysfunction and in that way influencing response. Patients with extrahepatic disease had a worse starting point compared to the patients who did not have extrahepatic disease at baseline when considering response. A trend test was used to evaluate a possible ordered relationship among the response categories. To determine discriminative capacity of tumour absorbed dose with regards to response, a receiver operating characteristic (ROC) analysis was performed [12]. Optimal thresholds for response on a tumour as well as a patient level (either CR or PR) was based on minimally 90% predictive value as this was deemed clinically relevant. Overall survival was investigated using Cox proportional hazard models penalized using Firth’s correction for small-sample bias [13]. The Cox proportional hazard model was adjusted for age, presence of liver cirrhosis, non-tumour dose and presence of extrahepatic disease at baseline. The assumption of proportional hazards in this model was not violated according to Schoenfeld residuals. Overall survival was analysed using landmark analysis to diminish time-to-response bias.

Analyses were performed using R statistical software, version 4.0.5 for Microsoft Windows accompanied by the following R libraries: coxphf (version 1.13.4), cutpointr (version 1.1.2), dplyr (version 1.1.4) ggplot2 (version 3.5.1), ggpubr (version 0.6.0), lme4 (version 1.1.35.3), nlme (version 3.1.160), pROC (version 1.18.5), readxl (version 1.4.3), survival (version 3.4.0), survminer (version 0.4.9), tidyr (version 1.3.1). Effect estimates are reported with 95% confidence intervals (CI) and corresponding 2-sided *p*-values.

## Results

Thirty-one patients with HCC were included (Table 1). Median time between baseline MRI and treatment was 14 days (range 1 – 53 days) and median time between treatment and first response assessment was days 90 (range 76 – 122 days). Three patients passed away before first follow-up imaging was scheduled, they were considered to have PD (both on tumour level as well as patient level). This resulted in a total of 80 lesions, of which 50 lesions were 5 mL or larger.

**Table 1:** Baseline characteristics of the HEPAR Primary patients (total *n* = 31). Qualitative data are numbers and percentages in brackets; continuous data are median and range in brackets

	*N* (%)
Gender	
Male	28 (90)
Female	3 (10)
Age (years, median (range))	73 (44–85)
BCLC stage	
Intermediate, B	9 (29)
Advanced, C	22 (71)
ECOG Performance status^$^	
0	18 (58)
1	13 (42)
Cirrhosis	20 (65)
Underlying liver disease^#^	
Alcohol aetiology	20 (65)
Hepatitis	
*B*	1 (3)
*C*	4 (13)
*NASH*^*&*^	3 (10)
Hemochromatosis	2 (4)
None of abovementioned	7 (22)
Child Pugh	
A5	18 (58)
A6	9 (29)
B7	4 (13)
Bilobar disease*	17 (55)
Portal invasion	6 (19)
Tumour thrombus	4 (13)
Non-tumour thrombus	1 (3)
Mixed type	1 (3)
Number of tumours	
1	4 (13)
2–3	4 (13)
3 >	23 (74)
Largest tumour diameter in mm (median (range))	56 (15–195)
Previous treatment^#^	
None	26 (84)
Resection	4 (13)
Ablation	4 (13)
TACE	1 (1)
Liver volume (mL)	1941 (1036–3460)
Treated volume (%)	54 (16–100)

^$^*ECOG* Eastern Cooperative Oncology Group Performance Status

^#^Some patients had more than one underlying liver disease/previous treatment

^&^*NASH* non-alcoholic steatosis hepatis

*Only LIRADS-5 lesions were considered

Median tumour absorbed dose (tumour level) was 95.5 Gy (range 44—332 Gy) and median mean tumour absorbed dose (patient level) was 116 Gy (range 46—303 Gy). Median target liver absorbed dose was 50 Gy (range 27—69 Gy). Median non-tumour absorbed dose based on whole liver volume was 19 Gy (range 3 – 48 Gy) and median non-tumour absorbed dose based on target liver volume was 30 Gy (range 13 – 54 Gy).

### Efficacy

**Fig. 1:**
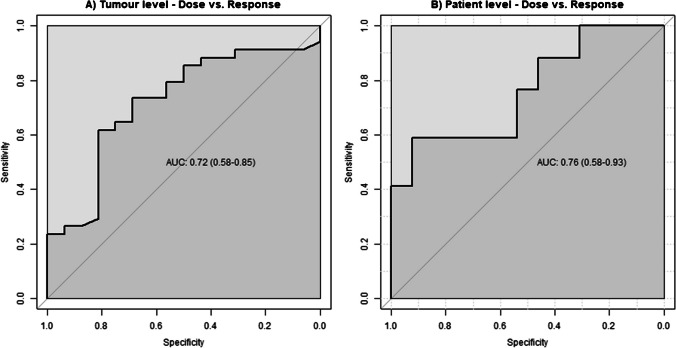
**A** Receiver operating characteristic curves (ROCs) showing discriminative value of tumour absorbed dose for response based on SPECT; AUC is 0.72 (95% CI: 0.58–0.85). **B** Ability of mean tumour absorbed dose per patient to discriminate between patients with complete or partial response and progressive or stable disease-based AUC is 0.76 (95% CI: 0.58–0.93). ROCs are not based on clustered data analysis, but area-under-the-curves (AUCs) are

**Fig. 2:**
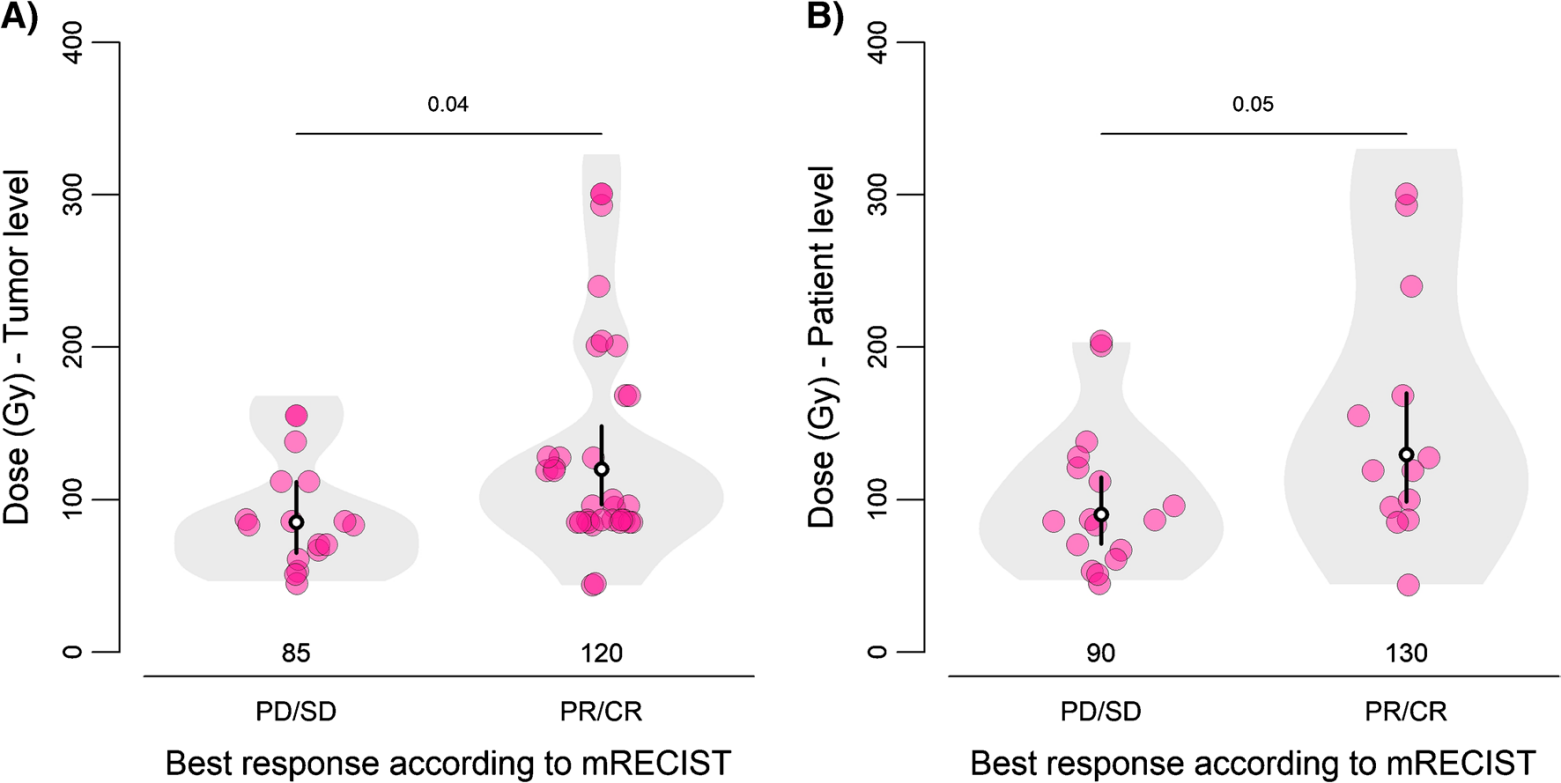
Relationship between (mean) tumour absorbed dose and best response according to mRECIST assessment on (**A**) **tumour level** (p for trend test is 0.0395) and (**B**) **patient level** (p for trend test is 0.0493). Average absorbed doses are indicated with white dot and black vertical lines represent 95% confidence intervals. *PD *progressive disease, *SD* stable disease, *PR* partial response, *CR* complete response

Median and ranges of absorbed doses per response category are presented in Table 2. Receiver-operating-characteristic (ROC) curves showed that the ability of tumour absorbed dose (tumour level) to predict response vs. non-response was 0.72 (95% CI: 0.58–0.85) (Fig. 1A). Tumour absorbed dose threshold with 100% positive predictive value for response at a tumour level was 184.5 Gy. At 90% predictive value the tumour absorbed dose threshold was 155 Gy (Table 3). The discriminative value of mean tumour absorbed dose (patient level) between responders and non-responders was 0.76 (95% CI: 0.58–0.93) (Fig. 1B). Mean tumour absorbed dose threshold for response at patient level with 100% positive predictive value for response was 222 Gy. At 155 Gy predictive value was 75% (Table 3). Responders received a 44% higher median absorbed dose compared to non-responders (range 1.9%-102.6%, p < 0.05, Fig. 2A)). Responding tumours received a 41% higher tumour absorbed dose compared to non-responding tumours (range 2.4%-93.3%, *p* = 0.04, Fig. 2B).

**Table 2:** Results of linear mixed-effects regression model showing percent change in mean absorbed dose per response category on both patient level (mean of the cumulative tumour absorbed doses) and tumour level

	Non-responders (PD + SD)	Responders (PR + CR)	P (trend)
Patient level	*n* = 9 + 8	*n* = 10 + 3	
Median (range) absorbed dose (Gy)	PD: 94 (53–147) SD: 108 (46–204)	PR: 127 (89–303) CR: 188 (199–299)	
Unadjusted	Reference	43.6% (1.9%-102.6%)	**0.05**
Adjusted*	Reference	22.5% (-16.7%-79.0%)	0.34
Tumour level	*n* = 4 + 13	*n* = 16 + 17	
Median (range) absorbed dose (Gy)	PD: 77 (53–147) SD: 83 (46–169)	PR: 118.5 (44–240) CR: 106 (46–332)	
Unadjusted	Reference	40.8% (2.4%-93.3%)	**0.04**
Adjusted*	Reference	24.1% (-12.1%-74.6%)	0.25

*Adjusted for non-tumour dose and presence of extrahepatic disease at baseline

*PD* progressive disease, *SD* stable disease, *PR* partial response, *CR* complete response

**Table 3:** (Mean) Tumour and Non-tumour absorbed dose thresholds with their respective sensitivity, specificity, positive predictive value, negative predictive value for response and liver toxicity* grade 3–5. *Liver toxicity grade 3–5 in either bilirubin and/or albumin levels and/or presence of ascites

	Threshold	Sensitivity	Specificity	Positive Predictive value (PPV)	Negative predictive value (NPV)
Tumour level response (tumour absorbed dose)	185 Gy	24%	100%	100%	38%
	155 Gy	26%	94%	90%	38%
Patient level response (mean tumour absorbed dose)	222 Gy	31%	100%	100%	65%
	155 Gy	46%	88%	75%	68%
Toxicity* grade 3–5 (non-tumour absorbed dose)	32.5 Gy	100%	14%	11%	100%
	17.5 Gy	67%	57%	14%	94%

### Toxicity

Laboratory and clinical adverse events up to six months after treatment can be found in the supplementary materials of Reinders et al*.* publication [6]. No cases of unacceptable toxicity as defined in the protocol were encountered. Grade 1–2 liver enzyme elevation was common and grade 2 or higher haematological toxicity was rare (besides lymphopenia).

**Fig. 3:**
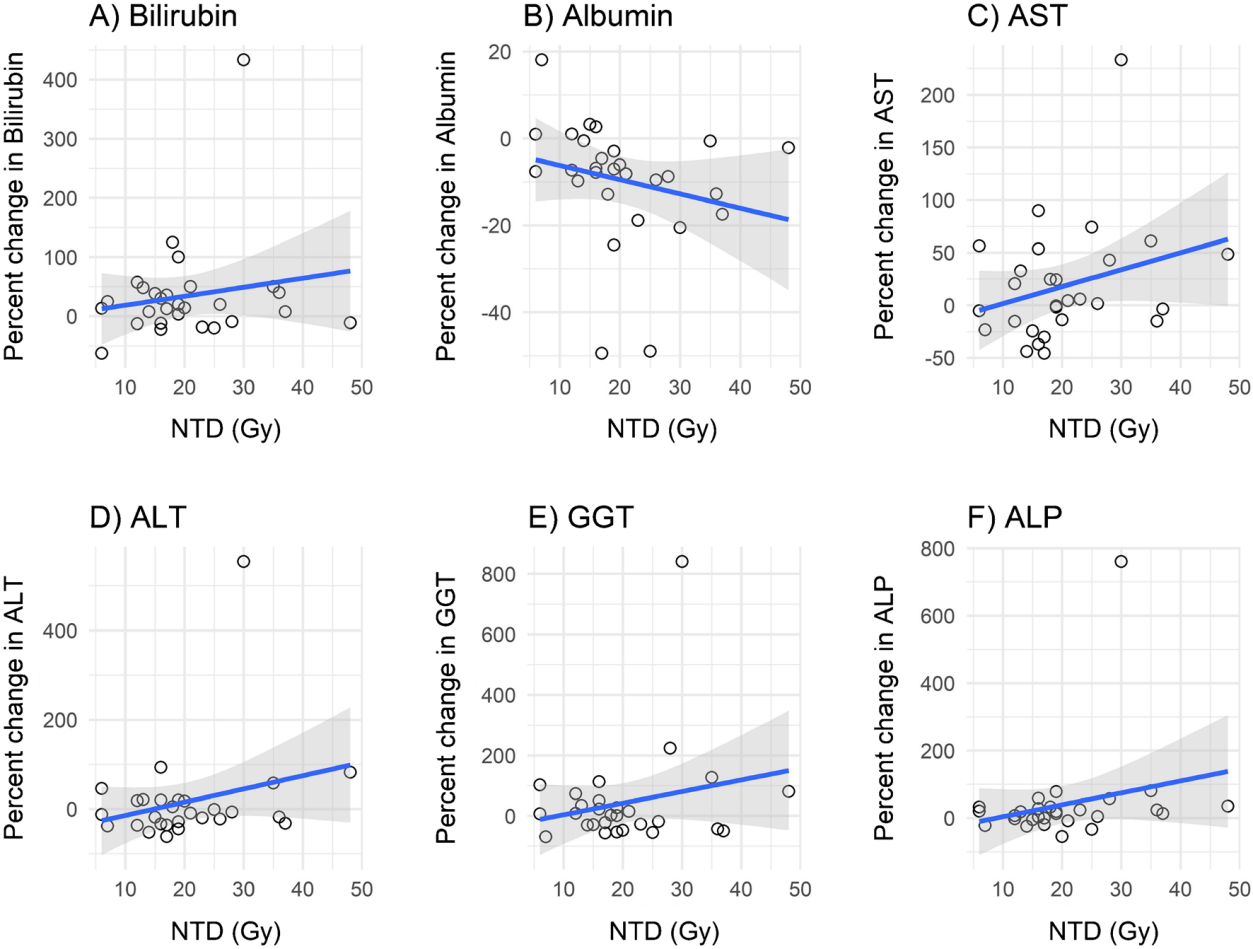
Association between change in laboratory values and non-tumour absorbed dose based on whole liver volume (NTD). Regression lines in blue and grey areas indicating 95% confidence intervals. *AST* aspartate aminotransferase, *ALT* alanine aminotransferase, *ALP* alkaline phosphatase, *GGT* gamma-glutamyl transferase

Linear regression analyses showed no significant relationship between non-tumour absorbed dose (Gy) based on the whole liver volume and percent change in bilirubin (3.21, 95% CI: -1.17–7.61, *p* = 0.15), albumin (3.73 (-0.80–8.25, *p* = 0.10), aspartate aminotransferase (AST) (-0.69, 95% CI: -5.54–4.16, *p* = 0.77), alanine aminotransferase (2.74, 95% CI: -3.56–9.03, *p* = 0.38), alkaline phosphatase (4.47, 95% CI: -1.68–10.62, *p* = 0.15) and INR (1.95, 95% CI:-5.18–9.09, *p* = 0.58). However, all graphs showed a trend towards an increase in percent change when non-tumour absorbed doses increased, except for albumin where an inverse relationship was observed (Fig. 3).

Linear regression analyses of non-tumour absorbed dose based on the whole liver volume against CTCAE graded laboratory adverse events showed that higher doses were associated with higher grade CTCAE toxicities in the unadjusted model, except for AST. Furthermore, a positive association was found between non-tumour absorbed dose based on whole liver volume and clinical toxicity, in particular nausea, vomiting, ascites and oedema of the limbs (Table 4). These same analyses were performed with the use of non-tumour absorbed dose calculated based on target volume (Figure S1 and Supplementary Table S2), showing similar results, albeit at lower significance, as expected. Three liver toxicity events grade 3–5 occurred. The discriminative value of non-tumour absorbed dose based on whole liver volume to distinguish between CTCAE grade 0–2 vs. 3–5 of liver toxicity was 0.54 (95% CI: 0.10–0.98). A 100% sensitivity for grade 3–5 bilirubin level and/or albumin level or ascites was observed at a non-tumour absorbed dose threshold of 32.5 Gy (Table 4).

**Table 4:** Association between non-tumour absorbed dose based on whole liver volume and laboratory adverse events according to CTCAE version 4.03. Significant *p*-values are in bold. For example, when looking at the unadjusted model entry for bilirubin one grade increase in toxicity is associated with a mean increase of 3.21 Gy in non-tumour absorbed dose

Variable (CTCAE v. 4.03 graded)	Number of patients with toxicity (*n* = 31)	Average change in absorbed dose (95% CI)	*p*-value	Corrected for mean tumour absorbed dose and patient response	*p*-value
Any laboratory AE	31	-0.63 (-9.06;7.79)	0.88	1.32 (-7.75;10.38)	**0.05**
Bilirubin	10	3.21 (-1.18;7.61)	0.15	4.19 (-1.33;9.71)	**0.02**
Albumin	20	3.73 (-0.80;8.25)	0.10	1.10 (-3.68;5.88)	**0.04**
INR	24	1.95 (-5.18;9.09)	0.58	2.85 (-4.36;10.06)	**0.04**
ALP	24	4.47 (-1.68;10.62)	0.15	4.93 (-1.29;11.14)	**0.02**
ALT	17	2.74 (-3.56;9.03)	0.38	2.36 (-4.43;9.14)	**0.04**
AST	29	-0.69 (-5.54;4.16)	0.77	1.95 (-4.08;7.97)	**0.04**
Child Pugh score		2.36 (-0.58–5.30)	0.11	1.22 (-2.01;4.45)	**0.04**
MELD score		0.29 (-0.93;1.50)	0.63	0.32 (-0.87;1.51)	**0.04**
ALBI grade		4.18 (-3.84;12.21)	0.29	0.86 (-7.37;9.09)	**0.05**
Any clinical AE	30	-0.09 (-1.67;1.50)	0.91	0.08 (-1.34;1.50)	** < 0.01**
Abdominal pain	10	0.99 (-2.31;4.30)	0.55	0.35 (-3.19;3.90)	** < 0.01**
Nausea	7	2.93 (-1.43;7.29)	0.19	-0.84 (-4.98;3.29)	** < 0.01**
Vomitus	2	9.79 (-0.10;19.68)	0.05	4.01 (-4.74;12.76)	** < 0.01**
Fatigue	22	-1.97 (-4.47;0.54)	0.12	-2.31 (-4.73;0.11)	** < 0.01**
Fever	4	0.13 (-7.51;7.76)	0.97	5.95 (-2.78;14.69)	** < 0.01**
Ascites	16	1.16 (-1.33;3.66)	0.36	1.76 (-0.87;4.38)	** < 0.01**
Hepatic failure	2	0.48 (-2.28;3.24)	0.73	0.95 (-1.44;3.34)	** < 0.01**
Dyspnoe**a**	6	-0.94 (-5.87;3.99)	0.71	-0.49 (-6.23;5.24)	** < 0.01**
Oedem**a** limbs	6	1.51 (-3.36;6.38)	0.54	9.01 (4.10;13.92)	** < 0.01**

## Survival

**Fig. 4:**
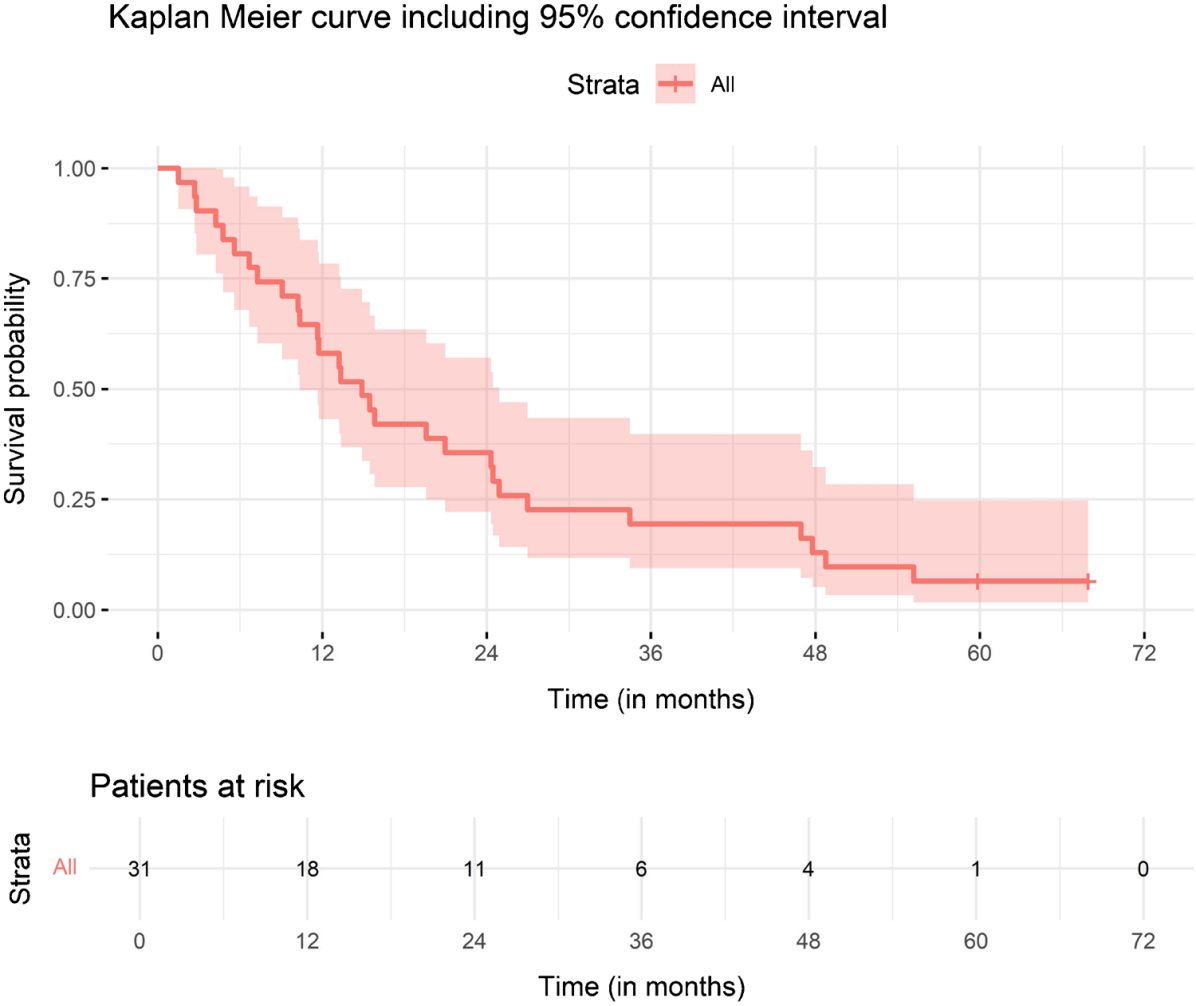
Overall survival of patients in HEPAR Primary study, median 14.9 months (95% CI: 10.4–24.9 months)

Survival data was censored on 17Jun2024, two patients are still alive. Overall survival was 14.9 months (95% CI: 10.4–24.9 months). Mean follow-up was 21.6 months (range 1.5 – 67.9 months) (Fig. 4).

## Discussion

The current study is the first prospective study on dosimetry after ^166^Ho-microspheres radioembolisation treatment in patients with HCC. Importantly, no cases of unacceptable toxicity were reported, and laboratory toxicities were generally low-grade. A positive association between higher tumour absorbed dose and better response both at a tumour level as well as on a patient level was observed. The same holds true for the association between higher non-tumour absorbed dose and higher grades of laboratory and clinical adverse events. These findings underscore the importance of individualized dosimetry for balancing efficacy and safety in HCC treatment with ^166^Ho-microspheres.

^166^Ho-microspheres and ^90^Y-microspheres are both used in radioembolisation, but these microspheres have distinct characteristics [14]. ^166^Ho-microspheres are paramagnetic, allowing post-treatment MRI imaging, which provides distribution analyses at higher resolution compared with PET or SPECT imaging [15]. They also emit gamma radiation detectable by SPECT imaging, enhancing distribution assessment at higher sensitivity, which allows for imaging at low activity levels, including a scout dose of ^166^Ho-microspheres as an alternative for ^99m^Tc-MAA. ^90^Y-microspheres primarily emit beta radiation (with a small branch of positron emission), which complicates imaging, especially at low activities [16]. Surrogate markers like ^99m^Tc-MAA for pre-treatment planning are needed, which may not perfectly match the therapeutic dose distribution. Post-treatment imaging for ^90^Y-radioembolisation requires PET or Bremsstrahlung SPECT.

Using a scout dose of ^166^Ho-microspheres offers significant advantages over ^99m^Tc-MAA in predicting lung shunt fraction and intrahepatic distribution of microspheres. Studies show that the distribution of a scout dose of ^166^Ho-microspheres closely mirrors the therapeutic ^166^Ho-microspheres distribution, providing more accurate predictions of lung shunt and intrahepatic distribution, crucial for minimizing non-target radiation and optimizing therapeutic efficacy [1]. Conversely, ^99m^Tc-MAA is less reliable in predicting the distribution of ^90^Y-microspheres, potentially leading to discrepancies in dose delivery [17]. Accurate lung shunt estimation is vital since high shunting can cause significant radiation exposure to the lungs, necessitating dose adjustments or leading to treatment contraindications [18].

Dose thresholds vary between tumour types, but also between types of microspheres used. The mean absorbed dose in a specific treated volume (i.e. tumour or non-tumour) can be equal, but the effect on tumour and non-tumour tissue can be completely different depending on the specific characteristics of the microspheres used. The main reason that mean absorbed dose values between the different types of microspheres cannot be directly compared is the difference in specific activity (i.e. the activity per microsphere) and the number of microspheres administered. A volume treated with a low number of microspheres (with a high specific activity) will show more heterogeneous distribution than a volume treated with a large number of microspheres (with a low specific activity). Non-tumour tissue is much more vulnerable if the absorbed dose is homogeneously spread, while a more homogeneous spread of microspheres in the tumour may theoretically be more effective [19]. The specific activity of ^166^Ho-microspheres is approximately 300–400 Bq/sphere, which is comparable to late week 2 glass ^90^Y-microspheres (i.e. approximately 200 Bq/sphere at day 12 after calibration) and 3–4 days pre-calibration resin ^90^Y-microspheres (i.e. approximately 200 Bq/sphere) [19, 20]. Since glass ^90^Y-microspheres are advocated to be used at lower number of microspheres (e.g. Wednesday week 1 up to Tuesday week 2), dose thresholds are higher for glass ^90^Y-microspheres than ^166^Ho-microspheres and dose thresholds are generally more comparable between resin ^90^Y-microspheres and ^166^Ho-microspheres [14].

Finding an optimal dose threshold is complicated as it needs to balance response of the tumour(s) on one side with the chance of developing high-grade toxicity on the other side. This study used 90–100% positive predictive value to establish the dose thresholds, where in literature 100% sensitivity, 100% specificity and highest overall accuracy have been used as well [21]. A high positive predictive value can be used as a ‘diagnostic tool’ in the planning phase of patient work up. It can be used as a target tumour absorbed dose above which chances for response are considerable and as a target non-tumour absorbed dose below which toxicity is assumed to be acceptable. Consequently, robust dose thresholds for an effective tumour absorbed dose and a safe functional liver-absorbed dose, especially for treatment subgroups, need to be refined and validated in larger series.

One of the limitations of this study was the limited number of patients included and the relatively heterogeneous patient and disease characteristics (e.g. presence of PVT, variety in target volume, number and size of tumours and as a result a large variety in treatment approach). As this was a phase I/II study and statistically powered to fulfil the primary endpoint of unacceptable toxicity, dosimetric endpoints and the ability to measure a potential relationship with response and toxicity were limited. Presented data and dose thresholds are therefore preliminary and should be validated and optimized in future studies. In the current study, radioembolisation treatment planning was performed according to a standard one-day treatment approach, in which all patients were intended to be treated with a 60 Gy absorbed dose to the target volume, regardless of tumour and non-tumour liver dosimetry. Individualised treatment planning was not used, while the option to make use of a ^166^Ho-microspheres scout dose is an advantage over ^99m^Tc-MAA [1]. The thresholds proposed in this study can support the use of a scout dose of ^166^Ho-microspheres in the future. Furthermore, treatment strategy varied among patients ranging between treating a few segments, one lobe or the whole liver. This difference in treatment strategy most probably impacted the dose-toxicity analyses. Non-tumour absorbed dose was therefore calculated based on whole liver volume as well as on target liver volume. Consequently, robust dose thresholds for an effective tumour absorbed dose and a safe non-tumour liver-absorbed dose, especially for these treatment subgroups, need to be refined and validated in larger series.

Therapies for patients with HCC are increasingly combined, for example immunotherapy after resection or ablation to decrease the chance of recurrence [20]. Combining therapies is expected to cause more toxicity in patients. Therefore, limiting radioembolisation adverse events and being able to predict the impact of absorbed dose on toxicity is of vital importance. The present study serves as a basis for dosimetric threshold finding, which can be used in personalised treatment planning for future HCC studies. The iHEPAR study (NCT05114148) will address this by studying the safety and efficacy of the implementation of personalised treatment planning based on specific dose thresholds in a prospective setting, similar to the current study. Additionally, exploring the use of ^166^Ho-microspheres in other liver malignancies and metastatic cancers could expand their therapeutic applications. Advances in imaging technology and dosimetry software may further enhance the precision and outcomes of radioembolisation treatments. Personalized treatment planning, considering patient-specific factors such as liver function and tumour characteristics, will be crucial in optimizing the use of ^166^Ho-microspheres in clinical practice.

In conclusion, this study demonstrated a positive dose–response relationship and dose-toxicity relationship for patients with HCC treated with ^166^Ho-microspheres radioembolisation. These findings can be used as a first step towards personalised treatment planning based on dosimetry.

## Supplementary Information

Below is the link to the electronic supplementary material.

Supplementary file1 (DOCX 1034 KB)

## Data Availability

No material was collected. Data is available upon request.

## Abbreviations

^166^Ho: Holmium-166
^90^Y: Yttrium-90
AASLD: American Association for the Study of Liver Disease
AE: Adverse event
AFP: Alpha-fetoprotein
CP: Child-Pugh
CTCAE: Common terminology criteria for adverse events version 4.03
ECOG: Eastern cooperative oncology group
HCC: Hepatocellular carcinoma
MELD: Model for end-stage liver disease
mRECIST: Modified response criteria in solid tumours
SAE: Serious adverse event
SPECT: Single photon emission computed tomography
TACE: Transarterial chemoembolization

## Code availability

R scripts are available upon request.
