# Supplementary material for: Holmium-166 radioembolisation dosimetry in HCC
Source: Eur J Nucl Med Mol Imaging. 2024 Oct 29;52(3):993–1003. doi: 10.1007/s00259-024-06940-2 (PMC11754330; doi:10.1007/s00259-024-06940-2)

# Supplementals

| Variable | Method | Number of patients with toxicity  grade 0-2 vs. 3-5 | Odds ratio  (95% CI); p-value | Corrected for tumour absorbed dose and patient response; p-value |
| --- | --- | --- | --- | --- |
| Any laboratory toxicity | NTD based on whole liver volume | 10 vs. 21 | 0.93 (0.45-2.01); 0.84 | 1.13 (0.45-3.20); 0.75 |
|  | NTD based on target volume |  | 1.18 (0.60-2.44); 0.64 | 1.61 (0.68-5.04); 0.26 |
| Any clinical toxicity | NTD based on whole liver volume | 21 vs. 10 | 1.03 (0.48-2.10); 0.94 | 1.18 (0.48-2.88); 0.85 |
|  | NTD based on target volume |  | 1.68 (0.84-3.70); 0.14 | 1.86 (0.88-4.53); 0.44 |

**Table S1:** The odds ratio given in this table represents the odds for encountering a grade 3-5 laboratory or clinical adverse event for every increase in 10 Gy of non-tumour absorbed dose based on whole liver and based on target volume.

| Variable  (CTAE v. 4.03 graded) | Number of patients with toxicity (n=31) | Average change in absorbed dose (95% CI) | p-value | Corrected for mean tumour absorbed dose and patient response | p-value |
| --- | --- | --- | --- | --- | --- |
| Any laboratory AE | 31 | 0.47 (-2.22;3.16) | 0.73 | 2.30 (-0.33;4.93) | **<0.01** |
| Bilirubin | 10 | 2.42 (-2.58-7.42) | 0.33 | 2.68 (-2.1-7.47) | 0.08 |
| Albumin | 20 | 5.44 (0.60-10.29) | **0.03** | 3.67 (-1.54-8.88) | 0.06 |
| INR | 24 | 4.68 (-3.10-12.46) | 0.23 | 4.33 (-3.15-11.8) | 0.08 |
| AF | 24 | 2.70 (-4.34-9.73) | 0.44 | 2.45 (-4.35-9.26) | 0.12 |
| ALAT | 17 | 0.29 (-6.82-7.41) | 0.93 | 0.22 (-6.71-7.16) | 0.14 |
| ASAT | 29 | -3.60 (-8.82-1.63) | 0.17 | -0.79 (-6.55-4.96) | 0.14 |
|  |  |  |  |  |  |
| Child Pugh score |  | 3.22 (0.16-6.27) | **0.04** | 2.77 (-0.03-5.86) | **0.04** |
| MELD score |  | 0.93 (-0.38-2.24) | 0.16 | 0.88 (-0.41-2.18) | 0.07 |
| ALBI grade |  | 8.58 (0.10-17.07) | **0.05** | 6.89 (-1.63-15.42) | **0.05** |
|  |  |  |  |  |  |
| Any clinical AE | 30 | 0.07 (-1.65;1.80) | 0.94 | 0.14 (-1.43;1.72) | **<0.01** |
| Abdominal pain | 10 | -2.16 (-5.78;1.47) | 0.24 | -2.19 (-5.50;1.10) | **<0.01** |
| Nausea | 7 | 0.68 (-4.13;5.50) | 0.78 | -2.27 (6.77;2.21) | **<0.01** |
| Vomitus | 2 | 9.32 (-1.58;20.22) | 0.09 | 4.39 (-5.70;14.49) | **<0.01** |
| Fatigue | 22 | -5.36 (-8.01;-2.71) | **<0.01** | -4.95 (-7.43;-2.49) | **<0.01** |
| Fever | 4 | -8.26 (-16.55;0.03) | 0.05 | -3.63 (-11.40;4.13) | **<0.01** |
| Ascites | 16 | 3.26 (0.56;5.97) | **0.02** | 2.99 (0.20;5.79) | **<0.01** |
| Hepatic failure | 2 | -1.27 (-4.30;1.76) | 0.41 | -1.30 (-4.05;1.45) | **<0.01** |
| Dyspnoea | 6 | -2.64 (-8.05;2.77) | 0.34 | -1.73 (-7.25;3.80) | **<0.01** |
| Oedema limbs | 6 | 0.88 (-4.47;6.23) | 0.75 | 4.66 (-0.42;9.75) | **<0.01** |

**Table S2:** Association between non-tumour absorbed dose based on target volume and laboratory adverse events according to CTCAE version 4.03. Significant p-values are in bold.

| Patient level  dose threshold (Gy) | Unadjusted | p-value | Adjusted* | p-value |
| --- | --- | --- | --- | --- |
| 88.5 | 0.56 (0.26-1.33) | 0.17 | 0.48 (0.18-1.47) | 0.15 |
| 95 | 0.50 (0.24-1.07) | 0.07 | 0.29 (0.12-0.76) | **0.03** |
| 131 | 0.85 (0.39-1.78) | 0.69 | 1.07 (0.44-2.53) | 0.32 |
| 155 | 0.84 (0.34-1.85) | 0.70 | 0.97 (0.35-2.51) | 0.31 |
| 222 | 1.28 (0.40-3.24) | 0.64 | 1.52 (0.45-4.33) | 0.28 |

**Table S3:** Results of Cox proportional hazard model per mean tumour absorbed dose threshold (patient level). *Adjusted for non-tumour absorbed dose, extrahepatic disease at baseline, presence of liver cirrhosis at baseline.

**Figure S1:** Association between percent change in laboratory values and non-tumour absorbed dose (NTD) based on target liver volume. Regression lines in blue and grey areas indicating 95% confidence intervals.


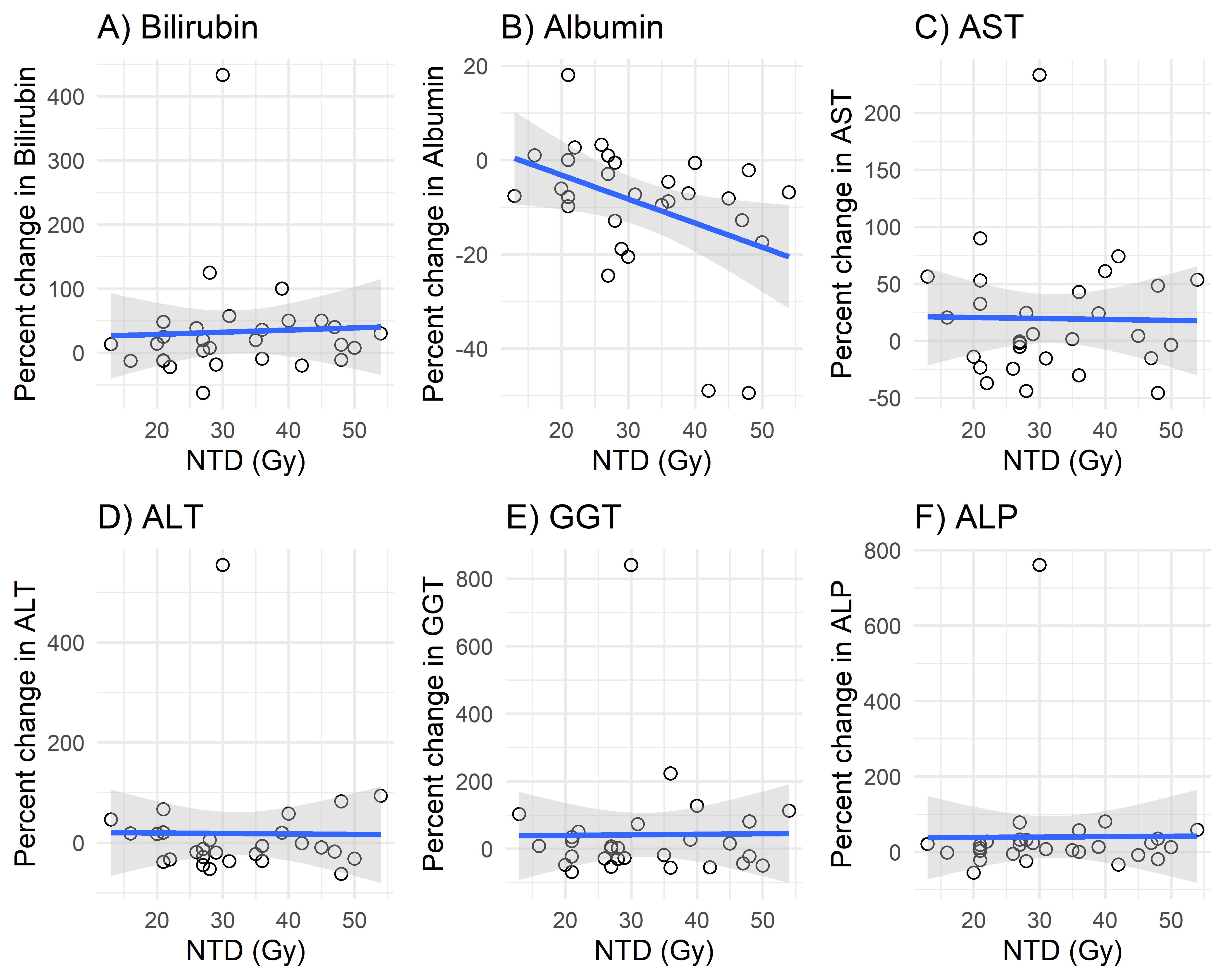

Supplement: Supplementary file 1 — Supplementary file1 (DOCX 1034 KB) [file 259_2024_6940_MOESM1_ESM.docx]
